# Adolescent motherhood in Bangladesh: Trends and determinants

**DOI:** 10.1371/journal.pone.0188294

**Published:** 2017-11-27

**Authors:** Mohammad Mainul Islam, Md. Kamrul Islam, Mohammad Sazzad Hasan, Mohammad Bellal Hossain

**Affiliations:** Department of Population Sciences, University of Dhaka, Dhaka, Bangladesh; University of West London, UNITED KINGDOM

## Abstract

**Background:**

While studies on fertility and contraceptives issues are available, until recently adolescent motherhood has not received enough attention among policy makers in understanding adolescent motherhood in Bangladesh. We aimed to examine the trends and determinants of adolescent motherhood among women aged 15–49 years.

**Methods:**

For trend analysis we used all the 7 waves of Bangladesh Demographic and Health Survey (BDHS, 1993–2014) data but for multivariate analysis 4 waves of BDHS (2004–2014). Two separate analyses were carried out on ever married women aged 15–49: (1) teenage girls aged 15–19 and (2) adult women aged 20 and above.

**Results:**

The prevalence of adolescent motherhood had declined to a slower pace from 1993 to2014 (from 33.0% to 30.8%). Lower spousal age gap and higher education were found to be associated with lower likelihood of adolescent motherhood both among teenage girls [OR 0.447 (0.374–0.533)] and adult women [OR 0.451 (0.420–0.484)]. Teenage girls in the poorest wealth quintile [OR 1.712 [1.350–2.173] were more likely to experience adolescent motherhood than the richest wealth quintile. Teenage girls who had no education were found to have 2.76 times higher odds of adolescent motherhood than their counterparts who had higher than secondary education. Concerning the time effect, the odds of adolescent motherhood among adult women was found to decline overtime.

**Conclusions:**

Despite substantial decrease in total fertility rate in Bangladesh adolescent motherhood is still highly prevalent though declining from 1993 to 2014. Social policies including those addressing poverty, ensuring greater emphasis on education for women; and adolescent mothers in rural areas are needed.

## Introduction

Adolescent motherhood is a major global problem due to the wide range of health effects and socioeconomic consequences both for mothers and their children, connected with it. Globally about 17 million adolescent girls give birth each year comprising 11.0% of all births worldwide. Majority of these births (95.0%) occur in low- and middle-income countries [1]. The prevalence of adolescent motherhood is much higher in low—income countries as compared to high income countries [1]. Half of all adolescent births occur in just seven countries: Bangladesh, Brazil, the Democratic Republic of the Congo, Ethiopia, India, Nigeria and the United States [1]. Bangladesh has the highest adolescent fertility rate in South Asia where 1 girl in 10 has a child before the age of 15 whereas 1 in 3 adolescent becomes mother or pregnant by the age of 19 [2–4]. Despite remarkable progress in human development adolescent childbearing is highly persistent in Bangladesh mostly due to the comparatively higher prevalence of child marriage [5, 6].

There are ample evidences suggesting that adolescent motherhood takes a toll on a girl's health, education and rights, which prevents her from realizing her own potential and has adverse impacts on the baby. Adolescent childbearing is generally associated with higher risk of adverse health outcomes of mother and newborn baby including a greater risk to maternal and child mortality [7, 8]. In addition, young mothers may not be sufficiently emotionally mature enough to bear the burden of childbearing [9]. Moreover, the economy of the country is also affected by early childbearing as adolescent mothers are not in a position to enter the workforce due to their double burden of household maintenance and child rearing [3]. Studies also show that women who marry at a young age are likely to find motherhood, at the expense of development in other areas including education, employment and personal development [5, 6].

A large body of research has looked at determinants of adolescent motherhood in the global context [3, 10, 11]. In general, findings of those researches show that early age at marriage, lower use of contraception, higher spousal gap, and lower socioeconomic attainment play a pivotal role in determining adolescent motherhood. Nevertheless, there are very few studies that have examined trends and determinants of adolescent fertility in Bangladesh [12–14]. Among recent studies, Kamal (2012) investigated factors affecting adolescent motherhood using data from the 2007 Bangladesh Demographic and Health Survey [14]. The author found that women’s education, husband’s education, place of residence, ever use of contraceptive method, religion, wealth and region are important determinants of adolescent motherhood in Bangladesh. Khan and Raeside (2007) noticed that adolescent girls who were living in rural areas or the followers of Islam (Muslim in religion) were more likely to have children before age 20 in Bangladesh[15]. Using data from the four waves of Bangladesh Demographic and Health Survey (1993–2004), Nahar and Min (2008) revealed that there were significant variations across divisions (larger administrative unit) in having first birth before age 20 among Bangladeshi women [13]. They showed that women’s higher education, higher age at marriage, and media exposure exerted strong influence in postponement of having children among adolescent girls in Bangladesh. It is evident that none of the earlier studies on adolescent motherhood in Bangladesh were done by using all (seven rounds) of the Bangladesh Demographic and Health Surveys [16–22]. Also those studies did not take into account ‘time’ effect in their analysis as important determinant to examine adolescent motherhood. This study is thus, an attempt to address these gaps in examining the trends and determinants of adolescent motherhood in Bangladesh.

The general objective of this study is to examine the trends and determinants of adolescent motherhood in Bangladesh. The specific objectives of this study are: (i) to examine the trends of adolescent motherhood pattern over times (1993–2014); and (ii) to investigate to what extent the factors influence adolescent motherhood among women in Bangladesh. To achieve the later objective this study follows two parts: the first part deals with teenage girls (aged 15–19) to examine their childbearing status and the second part aims to identify the determinants of adolescent childbearing among adult women aged 20 and above. We expect, this study will contribute towards better understanding of the dynamics of adolescent childbearing in Bangladesh and will also facilitate generating better policy directions to bring desired changes in adolescent childbearing in Bangladesh, which will eventually contribute to ensure quality life of adolescents and healthy life of mothers and children in particular.

## Data and methods

This study uses data from the Bangladesh Demographic and Health Surveys starting from 1993–94 to 2014. For trend analysis, all seven waves of data were used. However, for multivariate analysis four waves of data from the BDHS were examined: 2004, 2007, 2011 and 2014. Lack of identical information on wealth index precludes inclusion of data from waves prior to 2004. The analyses were done on at least once married women aged 15–49 at the time of the surveys. However, two separate analyses were conducted for two categories of women: (1) adolescent girls aged 15–19 at the time of the survey and (2) women aged 20 and above at the time of the survey to examine the childbearing status and the determinants of adolescent motherhood among teenage girls and adult women respectively. The total sample size for this study was 48,877 at least once married women aged 15–49. More specifically, the number of adolescent girls aged 15–19 was 1,489 in 2004 BDHS, 1,292 in 2007 BDHS, 1857 in 2011 BDHS, and 1970 in 2014 BDHS. Thus, the total sample size of teenage girls in four waves of the BDHS was 6,608. In addition, the number of adult women aged 20 and above was 8,167 in the 2004 BDHS, 7,879 in 2007 BDHS, 13,075 in 2011 BDHS, and 13,148 in 2014 BDHS. Therefore, the total sample size of adult women in four waves of the BDHS was 42,269.

### Variables

The outcome variable of interest in this study is adolescent motherhood. In this case, adolescent motherhood includes women who had either given first birth or were first pregnant before the age of 20. Several demographic, socioeconomic, cultural and spatial variables were included in the analysis as predictors of adolescent motherhood. Among demographic variables, spousal age gap was coded into three categories: less than five years, five to ten years, and more than ten years. Age of the respondents (in years) was included in the analysis as a continuous variable. Among socioeconomic variables, education (both respondents’ and partners’) was coded into four categories: no education, primary, secondary, and higher. Employment status of the respondents at the time of the survey was coded into two categories: employed and not employed. Wealth index was measured through five categories: poorest, poorer, middle, richer, and richest. Among cultural variables, religion (Islam or others) and access to media (yes or no) were included in the analysis. Respondents’ place of residence (rural versus urban) and division (Barisal, Chittagong, Dhaka, Khulna, Rajshahi, and Sylhet) were included in the analysis as spatial variables. Finally, a time variable (i.e., survey year) was included in the analysis to obtain unbiased estimates of the effects of independent variables on adolescent motherhood using data from the four time-points: 2004, 2007, 2011, and 2014.

### Analytical approach

Both bivariate and multivariate analyses were applied to explore the determinants of adolescent motherhood among ever married women in Bangladesh. Due to the dichotomous nature of the dependent variable multivariate logistic regression models were fitted to identify the demographic, socioeconomic, cultural and spatial determinants of adolescent motherhood. The results of the multivariate logistic regression analyses have been presented as odds ratios (OR) with 95% confidence intervals.

### Sample characteristics

Among the teenage girls, 68.3% had started childbearing before the age of 20. On the other hand, 78.6% of the adult women started childbearing before the age of 20. More than half of the respondents, in both categories, had spousal age gap of 5 to 10 years. The proportion of no education was lower among teenage girls compared to adult women. Among teenage girls, 10.5% were employed at the time of the survey compared to 23.5% in the case of adult women. The proportion of women in each category of wealth index for both categories of women were almost identical except in the case of the richest category where adult women had higher percentages than teenage girls. About 70.0% of the respondents in both categories lived in rural areas. Majority of the respondents were inhabitants of Rajshahi division which was followed by Dhaka, Chittagong, Khulna, Barisal and Sylhet. More than 90.0% of the respondents were followers of Islam. Adult women had higher media access than teenage girls (36.5% and 29.1% respectively). The difference in background characteristics between teenage girls and adult women were statistically highly significant. Among the respondents in both categories, mean age at first marriage was 15.0 years. The mean age at first birth was 15.0 years among teenage girls and 17.4 years among adult women (Table 1).

**Table 1:** Percentage distribution of women aged 15–19 and 20–49 in Bangladesh, 2004–2014.

Variables	Teenage Girls (15–19 Years) (N = 6,608)	Adult Women (20–49 Years) (N = 42,269)	Chi-square Test
**Had child before age 20**			
Yes	68.3	78.6	χ^2^ = 343.26, *p*<0.001
No	31.7	21.4	
**Spousal age gap**			
Less than 5 years	15.5	15.0	χ^2^ = 9.03, *p*<0.001
5–10 years	55.3	49.7	
More than 10 years	29.2	35.3	
**Education**			
No education	8.7	32.0	χ^2^ = 2245.71, *p*<0.001
Primary	27.7	31.7	
Secondary	57.3	31.3	
Higher	6.3	5.0	
**Employment Status**			
Employed	89.5	23.5	χ^2^ = 565.62, *p*<0.001
Not Employed	10.6	76.5	
**Wealth Index**			
Poorest	17.2	18.2	χ^2^ = 94.91, *p*<0.001
Poorer	21.4	19.0	
Middle	22.5	20.0	
Richer	21.6	21.0	
Richest	17.2	21.8	
**Education of Husband**			
No education	19.3	33.6	χ^2^ = 632.48, *p*<0.001
Primary	32.9	27.6	
Secondary	37.0	27.1	
Higher	10.8	11.7	
**Place of residence**			
Rural	69.8	66.5	χ^2^ = 26.36, *p*<0.001
Urban	30.2	33.5	
**Region**			
Barisal	12.9	12.4	χ^2^ = 20.29, *p*<0.001
Chittagong	17.7	16.5	
Dhaka	18.4	19.0	
Khulna	14.9	15.2	
Rajshahi	26.9	26.0	
Sylhet	9.4	10.9	
**Religion**			
Islam	92.0	90.1	χ^2^ = 23.18, *p*<0.001
Other	8.0	9.9	
**Media exposure**			
Yes	70.9	63.5	χ^2^ = 138.08, *p*<0.001
No	29.1	36.5	
**Survey year**			
2014	29.8	31.1	χ^2^ = 50.44, *p*<0.001
2011	28.1	30.9	
2007	19.6	18.6	
2004	22.5	19.3	
*Age (mean)*	*17*.*8*	*32*.*1*	
*Age at first marriage (mean)*	*15*.*0*	*15*.*0*	
*Age at first birth (mean)*	*15*.*0*	*17*.*4*	
*Spousal age gap (mean)*	*8*.*8*	*9*.*6*	
**N**	**6,608**	**42,269**	

### Ethics statements

The 2011 BDHS was conducted under the authority of the National Institute of Population Research and Training (NIPORT) of the Ministry of Health and Family Welfare. The survey was implemented by Mitra and Associates, a Bangladeshi research firm located in Dhaka. ICF International of Calverton, Maryland, USA, provided technical assistance to the project as part of its international Demographic and Health Surveys program (MEASURE DHS). An interview was conducted only if the respondent provided their verbal consent in response to being read out an informed consent statement by the interviewer. The permission of BDHS data set was available from https://dhsprogram.com/data and their user instructions were strictly followed (all BDHS data should be treated as confidential, and no effort should be made to identify any household or individual respondent interviewed in the survey). Ethical approval for the BDHS 2014 and 2011 was taken by the NIPORT from the BMRC (Bangladesh Medical Research Council).

## Results

### Trends of adolescent motherhood in Bangladesh: 1993–2014

**Fig 1:**
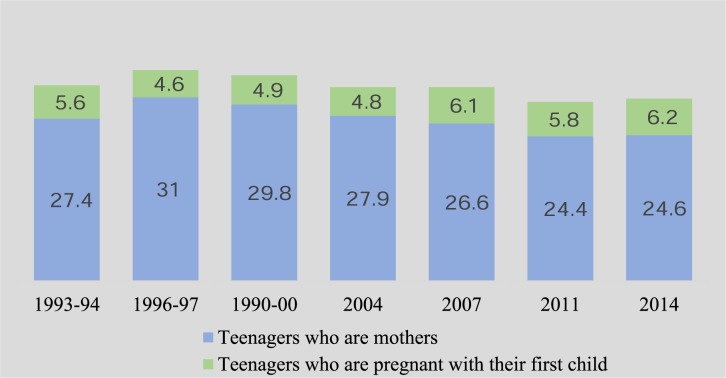
Trend in adolescent motherhood (%), BDHS 1993–2014.

Fig 1 presents trends in adolescent motherhood in Bangladesh during the period of 1993–2014. The rate of adolescent motherhood is presented in two categories: teenage girls who are mothers and teenage girls who are pregnant with their first child. It was found that the percentage of teenage mothers had declined from 1996–1997 to 2011. For instance, in 1996–97, 31.0% teenagers became mothers, which declined to 27.9% in 2004, and 24.6% in 2014.

On the other hand, there was no sustained pattern in the declining trend in the rate of pregnant with first child among teenage girls. For example, 1993–94, 5.6% teenagers were pregnant with their first child but it increased to 6.2% in 2011 with some fluctuations between 1993 and 2014. Overall, 33.0% teenagers had begun childbearing in 1993–94 which had decreased to 32.7% in 2004, and to 30.8% in 2014.

### Adolescent motherhood differentials among teenage girls by background characteristics

The percentage of teenage girls who started childbearing before the age of 20 had declined to some extent from 2004 to 2014 in Bangladesh. For instance, 11.5% teenage girls started childbearing in 2004 which had declined to 9.2% in 2014 (about 1 in 10). Similarly, the percentage of teenage girls who started childbearing by age 18 had declined to 31.1% in 2014 from 42.8% in 2004. Nevertheless, more than half of the teenage girls continued child bearing from the age of 18 onwards during the period of 2004–2014. The prevalence of adolescent motherhood was consistently lower in urban areas than rural areas during 2004–2014. In urban areas, more than one-fourth of the adolescent women became mothers whereas about one-third of the adolescent women became mother in rural areas (Table 2).

**Table 2:** Percentage of teenage girls (aged 15–19) who have begun childbearing, by background characteristics, Bangladesh 2004–2014.

Background Characteristics	Survey Year			
	2004	2007	2011	2014
**Teenager's age**				
15	11.5	11.4	9.8	9.2
16	22.2	18.6	16.1	16.2
17	37.2	33.4	28.9	31.1
18	42.8	42.5	38.8	41.4
19	58.8	58.5	58.3	57.8
**Place of residence**				
Urban	26.1	24.3	24.0	27.4
Rural	34.7	35.2	32.5	32.1
**Education**				
No education	45.7	48.4	46.7	48.3
Primary	43.0	42.1	39.4	44.3
Secondary	27.0	29.3	30.9	29.7
Higher	12.7	11.7	8.0	12.4
**Wealth Index**				
Lowest	42.8	41.5	41.6	41.1
Second	40.6	39.6	33.8	33.2
Middle	35.9	33.8	30.8	31.5
Fourth	29.8	33.5	28.6	28.0
Highest	18.6	19.7	19.3	22.9
**Region**				
Barisal	29.5	29.4	30.2	31.4
Chittagong	27.7	28.1	27.4	26.4
Dhaka	31.5	33.4	28.8	31.8
Khulna	37.7	33.9	32.9	31.2
Rajshahi	42.3	39.8	32.8	36.6
Rangpur	na	na	41.0	36.9
Sylhet	19.0	23.2	19.5	24.4
**Total**	**32.7**	**32.7**	**30.2**	**30.8**

The prevalence of adolescent motherhood was about four times higher among teenage girls with no education compared to those with higher than secondary education during the period of 2004 to 2014. Teenage girls in the lower wealth quintiles had substantially higher rate of adolescent motherhood than their counterparts in the higher wealth quintiles. There were also variations in adolescent motherhood across divisions. Among six divisions, teenage girls living in Rajshahi division had the highest rate of adolescent motherhood whereas Sylhet division had the lowest rate of adolescent motherhood during the period of 2004–2014 (Table 2).

### Adolescent motherhood differentials among adult women by background characteristics

The percentage distribution of adult women (aged 20–49) who had begun childbearing by age 20 is presented in Table 3. In this category, women aged 20–24 had the lowest rate of adolescent motherhood among the different age groups. Consistent with the prevalence of adolescent motherhood among teenage girls, the rate of childbearing before age 20 was found to be lower in urban areas than in rural areas. Similarly, the rate of adolescent motherhood among adult women was substantially higher among lower educated women. The inverse association between wealth index and the rate of adolescent motherhood is also clearly evident among adult women as well. In consistent with the earlier pattern, Sylhet division had the lowest rate of adolescent motherhood and Rajshahi division had the highest rate of adolescent motherhood among adult women (Table 3).

**Table 3:** Percentage of women age 20–49 who have begun childbearing, by background characteristics, Bangladesh 2004–2014.

Background Characteristics	Survey Year			
	2004	2007	2011	2014
**Age group**				
20–24	64.2	61.1	62.1	58.2
25–29	73.8	67.8	70.4	67.0
30–34	73.7	72.0	69.5	71.2
35–39	73.8	71.2	69.8	71.4
40–44	74.0	73.5	70.9	72.4
45–49	80.4	72.9	65.5	68.1
**Place of residence**				
Urban	63.9	59.0	59.0	56.6
Rural	74.5	71.5	70.9	71.6
**Education**				
No education	80.0	77.1	76.6	78.4
Primary	79.0	76.2	78.5	77.3
Secondary	63.9	62.9	64.2	65.9
Higher	17.2	18.5	16.5	16.1
**Wealth Index**				
Lowest	79.1	78.6	76.7	78.4
Second	78.7	73.7	74.9	74.2
Middle	76.0	72.4	71.7	72.6
Fourth	71.3	67.4	66.7	65.7
Highest	57.3	53.3	52.0	49.9
**Region**				
Barisal	72.5	73.0	69.2	66.3
Chittagong	67.8	65.4	65.2	65.1
Dhaka	71.2	66.8	64.7	65.7
Khulna	77.2	71.8	72.2	71.5
Rajshahi	76.4	73.8	72.1	71.5
Rangpur	na	na	76.8	75.0
Sylhet	60.7	55.6	53.2	55.4
**Total**	**72.0**	**68.6**	**67.7**	**67.2**

### Determinants of adolescent motherhood among teenage girls

Logistic regression estimates of the determinants of adolescent motherhood among teenage girls (aged 15–19) are presented in the form of odds ratios in Model 1 of Table 4. The model includes respondents’ spousal age gap, age, education, employment status, wealth index, place of residence, region, religion, exposure to media, husband’s education and time (year of surveys). Among teenage girls lower spousal age gap was found to be associated with lower risk of adolescent motherhood. For instance, teenage girls with less than five years of spousal age gap had 55.3% lower risk of adolescent motherhood than their counterparts with more than 10 years of spousal age gap after adjusting for the selected covariates. On the other hand, lower education was associated with higher odds of adolescent motherhood among teenage girls than higher education. More specifically, teenage girls who had no education were found to have 2.76 times higher odds of adolescent motherhood than their counterparts who had higher than secondary education. This inverse pattern was also true in the case of the effect of husband’s education on adolescent motherhood (Table 4).

**Table 4:** Odds ratios of adolescent motherhood among women in Bangladesh, 2004–2014.

Variables	Model 1: Teenage Girls (Aged 15–19 Years)	Model 2: Adult women (Aged 20–49 Years)
	Odds Ratio (95% C.I.)	Odds Ratio (95% C.I.)
**Spousal age gap**		
Less than 5 years	0.447** [0.374–0.533]	0.451** [0.420–0.484]
5–10 years	0.748** [0.654–0.854]	0.778** [0.734–0.822]
More than 10 years	[REF]	[REF]
**Age**	1.650** [1.577–1.724]	0.981** [0.977–0.983]
**Education**		
No education	2.764** [1.991–3.836]	5.265** [4.619–5.999]
Primary	2.842** [2.184–3.701]	5.589** [4.951–6.309]
Secondary	2.397** [1.904–3.018]	3.664** [3.294–4.075]
Higher	[REF]	[REF]
**Employment Status**		
Not Employed	0.856 [0.710–1.032]	1.049 [0.987–1.113]
Employed	[REF]	[REF]
**Wealth Index**		
Poorest	1.712** [1.350–2.173]	0.988 [0.890–1.096]
Poorer	1.482** [1.200–1.829]	1.009 [0.917–1.110]
Middle	1.318** [1.085–1.601]	1.017 [0.932–1.108]
Richer	1.288** [1.075–1.542]	1.037 [0.960–1.120]
Richest	[REF]	[REF]
**Education of Husband**		
No education	2.386** [1.861–3.059]	1.534** [1.384–1.699]
Primary	2.093** [1.692–2.59]	1.549** [1.408–1.705]
Secondary	1.478** [1.220–1.789]	1.309** [1.202–1.425]
Higher	[REF]	[REF]
**Place of residence**		
Rural	0.828** [0.724–0.947]	1.077* [1.016–1.413]
Urban	[REF]	[REF]
**Region**		
Barisal	0.908[0.709–1.163]	1.568** [1.422–1.729]
Chittagong	1.185[0.937–1.501]	1.557** [1.423–1.705]
Dhaka	0.992[0.786–1.251]	1.474** [1.350–1.609]
Khulna	1.087 [0.853–1.386]	1.908** [1.735–2.096]
Rajshahi	1.302* [1.039–1.630]	1.937** [1.777–2.109]
Sylhet	[REF]	[REF]
**Religion**		
Islam	0.977 [0.792–1.205]	1.415** [1.311–1.527]
Other	[REF]	[REF]
**Exposure to Media (Radio/TV/Newspaper)**		
Yes	0.767** [0.664–0.885]	0.995 [0.936–1.059]
No	[REF]	[REF]
**Survey year**		
2014	0.955 [0.813–1.122]	0.941 [0.873–1.012]
2011	0.878 [0.748–1.031]	0.899** [0.836–0.967]
2007	1.021 [0.857–1.216]	0.854** [0.788–0.925]
2004	[REF]	[REF]
**-2 Log Likelihood**	3698.482	20604.019
**N**	**6608**	**42,269**

*p<0.05

**p<0.01

Poorer wealth index was found to be associated with higher risk of adolescent motherhood among teenage girls compared to richer wealth index. For instance, Model 1 in Table 4 showed that teenage girls belonging to the poorest wealth quintile were 71.2% more likely to experience adolescent motherhood compared to their counterparts in the richest wealth quintile. Teenage girls living in rural areas were found to have 17.2% lower odds of adolescent motherhood than their counterparts living in urban areas. In addition, teenage girls living in Rajshahi division were found to have 30.2% higher risk of adolescent motherhood than those living in Sylhet division. Moreover, teenage girls having exposure to media were found to have 23.3% lower odds of having adolescent motherhood compared to those who did not have exposure to media (Model 1 in Table 4).

### Determinants of adolescent motherhood among adult women

Logistic regression estimates of the determinants of adolescent motherhood among adult women (aged 20–49 years) are presented in Model 2 of Table 4. In concordance with the findings of teenage girls, it was found that lower spousal age gap had lower odds of adolescent motherhood among adult women in terms of their age, education, employment status, wealth index, place of residence, region, religion, exposure to media, husband’s education and time (year of surveys). In addition, lower education was found to be associated with higher risk of adolescent motherhood among adult women. For example, adult women who had no education were 5.26 times more likely to become mothers before age 20 compared to those who had post- secondary education.

In contrast to the findings of teenage girls no significant difference was found in adolescent motherhood among adult women across various categories of wealth index. Adult women living in rural areas were found to have 7.7% higher odds of adolescent motherhood than their counterparts living in urban areas. Moreover, among adult women all divisions had significantly higher risk of adolescent motherhood than Sylhet division. More specifically, among women aged 20–49 living in Rajshahi division had the highest risk of adolescent motherhood (93.7%) followed by Khulna division (90.8%), Barisal division (56.8%), Chittagong division (55.7%), and Dhaka division (47.4%) compared to Sylhet division (Model 2 in Table 4).

## Discussion and conclusions

This study investigates the trends and factors affecting adolescent motherhood in Bangladesh using data from the Bangladesh Demographic and Health Surveys. The findings of the study suggest that despite substantial decrease in overall total fertility rate in Bangladesh, adolescent fertility is still highly prevalent. In addition, the trend of adolescent mothers who are pregnant with their first child had rather increased. The higher prevalence of motherhood among teenage girls can be attributed to the failure of preventing child marriage to a large extent in Bangladesh in one hand [5], and the lower rate of contraceptive use on the other [23]. It should be mentioned that though Bangladesh has made considerable progress in increasing overall contraceptive prevalence rate (44.6% in 1993–94 to 62.4% in 2014) it is still much lower among married teenage girls compared to adult women. For instance, in 2014 the prevalence of contraceptive use (any method) among teenage girls (aged 15–19) was only 51.2% compared to 67.7% among women aged 25–29. Part of the reason is that in most cases teenage girls take their desired number of children at younger ages to fulfill the expectation of husband and in-laws/ family members despite higher risk of having children before age 20 [24].

Higher spousal age gap both among teenage girls and adult women were found to be associated with higher adolescent motherhood. Possible reasons might be that higher spousal age gap leads to unequal power relations in the family and low level of inter-spouse communication which essentially translate into women’s lower participation in family decision making process including decision to use contraceptives [25, 26].

Regarding women’s education and adolescent motherhood an inverse association was found. This was also true in the case of the impact of husband’s education on adolescent motherhood. Plausible explanations of the inverse association between educational attainment and adolescent motherhood could be that lower educated women have lack of adequate knowledge about the high risk period of becoming pregnant, are not fully aware about family planning methods and the negative consequences of early childbearing on their health and children as well [27, 28]. These factors are further aggravated by the fact that lower educated women have lower levels of empowerment within family and society which eventually translate into higher level of adolescent motherhood [29, 30].

Similar to education, poorer wealth indexes were found to be associated with higher prevalence of adolescent motherhood among teenage girls. This can be explained by the facts that the rate of child marriage is higher among poor females which eventually leads to early childbearing among millions of young females in Bangladesh since there is a social pressure of having children in the first year of marriage [31]. In most cases, these young females are characterized by lower educational attainment, lack of adequate knowledge about the negative consequences of early childbearing are financially dependent on their husbands and limited to no role in the decision making process which essentially restricts their capability to postpone their childbearing to older ages [31]. In addition, exposure to mass media were found to exert strong positive impact in reducing adolescent motherhood among teenage girls. Exposure to media contributes to creating awareness about negative consequences of early childbearing and are very influential in motivating couples in adopting family planning methods [32, 33].

Two contrasting findings were revealed concerning religion: among teenage girls there was no significant difference in adolescent motherhood between Muslims and the followers of other religion. However, among adult women, Muslims were found to have higher adolescent motherhood than the followers of other religion. These contrasting findings about the impact of religion on adolescent motherhood can be explained by the fact that the role of religion on various aspects of personal life in general and on fertility behavior in particular has been diminishing over time due to the impact of modernization, urbanization [34]. Similar to the effect of religion, another contrasting finding was that teenage girls living in rural areas had lower adolescent motherhood than those living in urban area whereas adult women in rural area had higher adolescent motherhood than urban areas. Sample size variations among the two groups might be one reason. However, another possible explanation might be the positive impact of large scale socioeconomic development, access to electronic media and family planning interventions taken by the Government and NGOs in Bangladesh. It can be mentioned that a large number of NGOs are working in rural areas for overall socioeconomic development in Bangladesh for the last few decades. In addition, substantial variations were found in adolescent motherhood across divisions among adult women in Bangladesh which can be due to the differences in the prevalence of poverty, child marriage, and contraceptive use across divisions. Concerning the time effect, the odds of adolescent motherhood among adult women was found to decline overtime after adjusting for the selected covariates. The effect of time on adolescent motherhood can be attributed to the increasing trend of female education, labour force participation, women empowerment, and knowledge dissemination in Bangladesh. In connection with this it is worthwhile to mention that Bangladesh is one of the few developing countries that has achieved most of the Millennium Development Goals including reducing poverty, increasing female education, and reducing gender inequality [35, 36].

Our study is not devoid of limitations. For this study to examine the factors affecting adolescent motherhood we analyzed only the BDHS 2004–2014 data rather than all other previous data. Also the determinants of adolescent motherhood could be classified as proximal and distal which are not shown in this study. Also, this study does not have a qualitative component and therefore does not aim to give an in-depth analysis of the changes in the incidence of adolescent motherhood.

In conclusion, it can be argued that adolescent pregnancy is the result of diverse underlying societal, economic and other forces, preventing it requires multidimensional strategies that are oriented towards girls’ empowerment and tailored to particular populations of girls, especially those who are marginalized and most vulnerable [3]. Some of the recommendations provided here based on the findings of our study are: (i) addressing poverty can increase the age at marriage and resist adolescent motherhood. Policymakers should support interventions at the national and regional levels where age at marriage is low and adolescent motherhood is high, that economically empower girls and women; (ii) for stopping early marriage and early pregnancy the current emphasis on education for women(keeping girls in school) should be continued; special protection to poorer girls for enabling them to continue education and facilitate their participation in formal employment are needed; (iii) increasing adolescents’ access to sexual and reproductive health, including contraception should be ensured; (iv) provision of better support to adolescent mothers based on regional variations should be arranged; and (v) in-depth studies are needed to explore the vulnerabilities of adolescent women to understand adolescent motherhood dynamics in Bangladesh.
